# Obstetrics and Gynecology

**Published:** 1895-01

**Authors:** 


### OBSTETRICS AND GYNECOLOGY.

EDITED BY DR. VIRGIL O. HARDON.

### TRANSVERSE POSITIONS OF THE VERTEX IN THE INFERIOR STRAIT OF THE PELVIS.

Dr. Maurice Muret: The *résumé* of this very interesting paper is as follows: *Etiology.*—(1) Primary transverse positions occur in non-rachitic but flattened pelvis when the fetal head is greatly developed (pelvis having a double congenital dislocation of the femurs); in flattened and generally narrow pelvis; funnel-shaped pelvis "*en entonnoir*"; large pelvis and small head. (2) Secondary transverse positions occur in: occipito-posterior presentations arrested during their rotation; large heads; insufficiency of pains. (3) Mixed cases: flattened pelvis and occipito-posterior presentation arrested during rotation.

*Evolution—Rotation can be finally accomplished* at the inferior strait or in the muscular part of the pelvis. In both cases spontaneous delivery the head is in the occipito-pubic, more rarely fronto-pubic presentation.

*Rotation cannot take place* on account of arrest in labor from insufficient pains; very strong pains, producing a sort of wedging in of the head with edema of the vulva; the head descends from the bony pelvis into the soft pelvis in a transverse position; the head disengages transversely.

*Diagnosis.*—Measurements of pelvis important. In primary transverse positions the O.I.S. left occipito-iliac are most frequent; in the secondary, the position is always O.I.D. The relative height of fontanels variable; the greater fontanel is low down, more commonly in secondary than in the primary positions; in the latter position the small fontanel is more frequently low (flattened and generally narrow pelvis).

*Prognosis.*—Relatively favorable for both mother and child. However, there is danger of vaginal fistula, large lacerations of the perineum, rupture of pelvic articulations for the mother; for the child, long duration of the accouchment, compression of the head, intracranial hemorrhages.

*Treatment.*—This should be expectant as long as possible. Hygienic measures. Lateral ducubitus. Stimulants to fortify the patient and bring on the pains. Uterine expression according to Kristeller's method. Tarnier's manual method. When indicated, the forceps should be applied in one of the oblique diameters of the pelvis. Never apply them in the antero-posterior diameter! Excepting in cases of absolute necessity, never apply the forceps on the occipito-frontal diameter of the head. Do not use forced rotation with the forceps. Simple traction. Once the head in the soft pelvis, remove the forceps. In cases of transverse position in the soft pelvis, employ my manual method for correcting the position.* Perforation of the head is rarely necessary. If the child is alive,

*Muret's method is as follows: Two fingers of the right hand are introduced into the vulva and the small fontenel is searched for. This is usually found immediately above one of the sciatic tuberosities, and aided by the projection formed by the union of the lambdoid and sagittal sutures, the occiput is brought forward, while with two fingers of the left hand in the rectum, the forehead is at the same time pushed towards the perineum. This maneuvre is accomplished without difficulty and the head disengages rapidly.

and if the disproportion is not too great, do symphyseotomy preferably. If the child is dead, perforation is to be preferred to a difficult delivery with the forceps.—*Nouvelle Arch. d'Obstet. et Gyenécol.*, May and June, 1894.

---

### GONORRHEAL SALPINGITIS.

Walton (*Centralblatt für Gynäkologie*), in a very complete monograph on this subject, refers to a latent form of gonorrhea, where the organism is saprophytic, and which exists in the male as well as the female. This latent organism, through an exciting cause, where a favorable culture medium is present, manifests itself, and thus it is that in the female the different uterine and adnexia imflammations are brought about. A pyosalpinx is commonly caused by a mixed infection of gonococci and pyogenic organisms, and this mixed infection can only be found early in the case, since the gonococci are later overpowered by the pus cocci. The gonococci tend to migrate from the vagina into the cervix, and thence into the uterine cavity. The tubes are infected by uterine contraction or some therapeutic measure. German statistics show twenty-three to twenty-eight per cent., and English and American seventy per cent. of all cases of adnexia disease to be due to gonorrhea. The inflammation always extends to the ovary, and may cause here a variety of conditions, from an oophoritis to an abscess, and, also salpingitis is always associated with perimetritis, *i. e.*, the inflammation extends through the tube wall, causing a perisalpingitis, or else direct through the tube (ostiuma bdominalis), infecting the peritoneum, and at times causing general peritonitis. Usually the inflammation does not extend beyond the broad ligament, or where there is a pyosalpinx it may infiltrate between the two peritoneal layers of the same. The entire tube is never involved, but the inflammation is limited to the outer two-thirds, where the mucous membrane folds are more complex, and the growth of the microorganisms more favorable. Walton believes these cases should be treated antiphlogistically at the beginning, and later by hot irrigation ($109\frac{2}{5}$° to 113° F.), followed by an iodoform or ichthyol-glycerine tampon. He follows the operative technique of Lawson Tait, except where there are numerous adhesions. If hemorrhage, or the pus has escaped into the abdominal cavity, he irrigates with warm water at a temperature of 104° F., where Tait uses water at $98\frac{3}{5}$° F. In these cases he also uses a glass drainage tube.—*University Medical Magazine.*

---

### TREATMENT OF VOMITING OF PREGNANCY.

Lutaud states that vomiting of pregnancy is best treated by cocaine. The action of this drug is often strengthened by combining it with antipyrin. Thus the following prescription:

| | | |
|---|---|---|
| ℞ | Chlorohydrate of cocaine | gr. iss. |
| | Antipyrin | gr. xvi. |
| | Distilled water | ℥ iv. |

Sig. One teaspoonful every half hour until vomiting ceases.

If the stomach will not tolerate this quantity of liquid, 10 drops of a one and a half or two per cent. solution of cocaine are administered, repeated at one or two hour intervals.

At times the application of cocaine to the os is extremely valuable.

The following prescription may be used:

| | | |
|---|---|---|
| ℞ | Hydrochlorate of cocaine | gr. xvi. |
| | Extract of belladonna | gr. iv. |
| | Vaseline | ℥ ss. |

Cotin's method of dilating the os with the finger sometimes causes immediate cessation of vomiting. Occasional success will follow Routh's procedure, which consists in exposing the uterine neck by means of a speculum and painting with tincture of iodine. In cases of moderate severity, the following mixture will be found serviceable:

| | | |
|---|---|---|
| ℞ | Tincture of iodine | ℥ ii. |
| | Chloroform | ℥ ii. |

Sig. Five drops night and morning at meal times, taken in Seltzer water.

—*Annals of Gynecology.*

---

### SYPHILIS AND PREGNANCY.

Fournier believes that two of the most important factors in the diagnosis of hereditary syphilis in a family are great frequency of abortion and high infantile mortality. Abortion is least frequent when the father alone is syphilitic, more frequent when the mother alone is syphilitic, and most constant when both parents are affected. In the latter cases as many as nineteen abortions have been known to occur. Fournier attended a family in which the first three children were all born at term and all robust. Then the father contracted syphilis, and his wife became affected ; she aborted three times in succession.

Fournier found that at the Lourcine Hospital 145 out of 167 of the children born of syphilitic mothers died in the institution. Collecting trustworthy statistics of 441 cases reported elsewhere, 100 children whose mothers were syphilitic survived infancy, while 341 died. It is noteworthy that out of the 341 that died, 335 perished within their first year; only six died later. Out of nine children in a syphilitic family, only two are likely to survive their first year.—*Univ. Med. Mag.*

---

### TREATMENT OF VULVO-VAGINAL VEGETATIONS BY CAUTERIZATIONS WITH PURE CARBOLIC ACID.

Dr. Raulin says he has treated with success vulvo-vaginal vegetations of considerable extent by this method, formerly employed by Tomaso de Amicis, Jullien, and Derville. The treatment is as follows: After carefully washing and cleaning the parts, the bottle containing the carbolic acid is heated either by a flame or by placing it in boiling water until the substance is liquefied. A piece of absorbent cotton dipped in the acid is passed over the entire surface of the vegetation. The color of the tumor turns immediately from the pink that it was, to a dull white. This layer is eliminated after two or three days, or is removed by washing, and another application is made in the same way. The neighboring parts are covered with vaseline, so as to protect them from the acid. The applications are entirely free from pain, and even if there were, a little cocaine will be all that is necessary. The therapeutic effect of these applications is sure and rapid. In one of the writer's cases three applications were quite sufficient.—*Journal de Méd. de Bordeaux*, review in *Journal de Méd. et Chirurgie pratiques*, July 10, 1894.

